# Completion of Volume Four

**Published:** 1865-07

**Authors:** 


### EDITORIAL DEPARTMENT.

### COMPLETION OF VOLUME FOUR.

The fourth volume of our Journal closes with our present issue, and it is but natural that we intrude upon its readers some facts and reflections pertaining to its personal history—its objects, condition and prospects. Four years since the Buffalo Medical and Surgical Journal was commenced, with the view that Buffalo was a medical centre of importance, and that to it would naturally be directed the interest and sympathy of a large circle of professional influence and support from western New York, and the adjoining States; that Buffalo itself sustained a Medical College of repute and influence, affording unsurpassed advantages and attractions; has hospitals, asylums, dispensaries, &c., &c., furnishing abundant fields for observation, with a living, active, earnest profession, ambitious to discover medical truth, and advance and extend medical science. To how great an extent this view has been sustained, and the just expectations of the friends of this enterprise realized, we shall leave our readers to judge; suffice it for us to say, that through the indulgence and favor of the profession, the Journal has been thus far generously sustained, and, with no just ground of complaint, and with many reasons for gratitude, we hope to continue in an enterprise which, though it has yielded no profit, and cost great labor, has yet been productive of benefits which are inseparable from earnest working.

The object in publishing this Journal has been to extend the influence of correct observation, to separate truth from error in medicine, and to present that only which is valuable; opposing error and ignorance with the only legitimate weapons of science and truth. It has had no private or partial interests to promote; it is dependent upon no special favor, but asks the support and indulgence of all. It has for its object the collection and diffusion of medical knowledge, and is open for the publication of all honest and intelligent observation.

The history of this journal commences with the history of a rebellion unequaled in magnitude, unparalleled in barbarity, and unrivaled in its achievements for progress and civilization. It has been sustained through this period of commercial and political revolution, and now, in the calm of restored peace, indulges hopes of still more liberal support, more hearty sympathy, more extended usefulness. It will be conducted as heretofore in the interest only of the profession, and will rely upon it for countenance and support. Cordial invitation is again extended to the profession to favor the journal with original communications, and hearty thanks are returned for the favors of the past. The journal, as will be observed, is gradually growing in the number of its pages, as well as in the practical value of its contents. This increase in the pages of the journal has been in obedience to a personal ambition on the part of the editor, rather than to any inherent ability with the journal itself. It is the design to furnish its readers with the greatest amount of the best possible material, which the resources of the journal will permit, deficiencies to be made up from independent sources.

Restored peace has indeed brought us much for which to be thankful, but reduction in the price of publishing our journal is not included in the list, though some improvements in its style have already been gained. We hope to see the time when the present subscription price of the Journal will allow a greatly increased number of pages, and other improvements too numerous to mention; meanwhile, we would say to our readers, please invite all your acquaintances to forward their names as subscribers to which *Volume Five* commences with our next issue.

We hope to hear from some of our old subscribers who have too long forgotten us, before they are greeted with the first number of the next volume. We are modest in this invitation, not because the printer is paid or can afford to wait, but because so few have neglected us, and so many of the profession both in the city and country have not only satisfied all just claims, but have in various ways placed us under lasting obligations, so that the debt of gratitude which we owe is so immeasurably greater than obligations which are due, that we scarce venture to intimate any delinquency whatever on the part of others. Our friends in distant places who have formed acquaintance with us mainly through the pages of the Journal, and have so frequently and generously manifested their friendship and professional confidence, will please accept our thanks, and an assurance that nothing could yield us so much gratification, or constitute so high an object of ambition, as to be able to really merit these favors. Cheered, sustained, and stimulated by the success of our journal in the past, we shall commence the duties of the future with fuller confidence and livelier hope, believing that our readers will sustain us when we are right, and correct and forgive us when wrong.

